# Tumor cell-macrophage interactions increase angiogenesis through secretion of EMMPRIN

**DOI:** 10.3389/fphys.2013.00178

**Published:** 2013-07-12

**Authors:** Bat-Chen Amit-Cohen, Maya M. Rahat, Michal A. Rahat

**Affiliations:** Immunology Research Unit, Carmel Medical Center and the Ruth and Bruce Rappaport Faculty of Medicine, Technion-Israel Institute of TechnologyHaifa, Israel

**Keywords:** membranal and secreted EMMPRIN, VEGF, MMP-9, tumor cells, macrophages, angiogenesis, mir-146a

## Abstract

Tumor macrophages are generally considered to be alternatively/M2 activated to induce secretion of pro-angiogenic factors such as VEGF and MMPs. EMMPRIN (CD147, basigin) is overexpressed in many tumor types, and has been shown to induce fibroblasts and endothelial cell expression of MMPs and VEGF. We first show that tumor cell interactions with macrophages resulted in increased expression of EMMPRIN and induction of MMP-9 and VEGF. Human A498 renal carcinoma or MCF-7 breast carcinoma cell lines were co-cultured with the U937 monocytic-like cell line in the presence of TNFα (1 ng/ml). Membranal EMMPRIN expression was increased in the co-cultures (by 3–4-folds, *p* < 0.01), as was the secretion of MMP-9 and VEGF (by 2–5-folds for both MMP-9 and VEGF, *p* < 0.01), relative to the single cultures with TNFα. Investigating the regulatory mechanisms, we show that EMMPRIN was post-translationally regulated by miR-146a, as no change was observed in the tumoral expression of EMMPRIN mRNA during co-culture, expression of miR-146a was increased and its neutralization by its antagomir inhibited EMMPRIN expression. The secretion of EMMPRIN was also enhanced (by 2–3-folds, *p* < 0.05, only in the A498 co-culture) via shedding off of the membranal protein by a serine protease that is yet to be identified, as demonstrated by the use of wide range protease inhibitors. Finally, soluble EMMPRIN enhanced monocytic secretion of MMP-9 and VEGF, as inhibition of its expression levels by neutralizing anti-EMMPRIN or siRNA in the tumor cells lead to subsequent decreased induction of these two pro-angiogenic proteins. These results reveal a mechanism whereby tumor cell-macrophage interactions promote angiogenesis via an EMMPRIN-mediated pathway.

## Introduction

Solid tumors include tumor and stroma cells, particularly infiltrating macrophages, which may consist of up to half of the tumor mass (Mantovani et al., 2002; Murdoch et al., 2004). Macrophages can be activated to become effector killer cells (classically or M1 activated) or to be involved in wound healing and angiogenesis (alternatively or M2 activated) (Mosser and Edwards, 2008; Gordon and Martinez, 2010). However, since we now realize that macrophages are very plastic and can be activated in many additional modes according to the microenvironment, this dichotomy is really a simplified way to describe their activation and function.

The three main macrophage subsets that are recognized within the tumoral context are tumor-associated macrophages (TAMs) (Lewis and Murdoch, 2005), Tie2 expressing monocytes (TEMs) (De Palma et al., 2007), and myeloid-derived suppressor cells (MDSCs) (Serafini et al., 2006; Murdoch et al., 2008), all of which secrete varying amounts of pro-angiogenic factors (e.g., vascular endothelial growth factor-VEGF), anti-inflammatory mediators (e.g., TGF-β, IL-10, IL-13, PGE_2_) that inhibit the tumoricidal activity of immune cells (Mantovani et al., 2004; Murdoch et al., 2005), and enzymes that degrade the extracellular matrix (ECM) and make room for the growing tumor mass (e.g., matrix metalloproteinases-MMPs). MMPs and VEGF are both crucial for tumor progression, invasiveness, metastasis and angiogenesis (Egeblad and Werb, 2002). High amounts of MMPs, particularly MMP-9, degrade the ECM, release and activate VEGF, and allow migration of cells (including infiltration of leukocytes, spreading of metastatic tumor cells, and integration of pericytes and endothelial cells into the tumor vasculature) (Murdoch et al., 2008). VEGF is an extremely potent pro-angiogenic factor, a chemoattractant for macrophages, and a regulator of MMP-9 (Owen et al., 2003). Thus, a regulatory positive loop exists, where MMP-9 regulates VEGF bioavailability, and VEGF regulates MMP-9 expression (Hollborn et al., 2007).

Thus, the tumoral microenvironment is rich in anti-inflammatory mediators (e.g., IL-10, IL-13, TGFβ, PGE_2_) that skew macrophages toward alternative/M2 patterns of activation, while hypoxia immobilizes them at the site (Murdoch et al., 2004). However, the microenvironment also consists of low levels of pro-inflammatory cytokines (e.g., IL-1β, TNFα) which paradoxically enhance tumor angiogenesis and proliferation (Balkwill, 2009). TNFα in particular can be produced by the tumor cells not only to enhance their proliferation, but also to promote invasiveness through its ability to induce macrophage MMP-9 (Hagemann et al., 2004)

In many solid tumors, expression of VEGF and MMPs, including MMP-9, is up-regulated by the extracellular matrix metalloproteinase inducer (EMMPRIN, also called basigin or CD147). This is a multifunctional protein that is expressed on the surface of both tumor and stroma cells, including macrophages (Yan et al., 2005; Nabeshima et al., 2006), and through homophilic EMMPRIN:EMMPRIN interactions between these two cell types may induce the expression of both MMPs and VEGF and increase angiogenesis (Tang et al., 2004, 2005; Yurchenko et al., 2010). Other protein partners for EMMPRIN, such as cyclophilin A and B, may also be responsible for its pro-angiogenic activity, as well as for inducing chemotaxis and recruitment of leukocytes to the tumor (Yurchenko et al., 2010). Overexpression of EMMPRIN was found in many types of tumors, and was correlated to VEGF and MMP-9 induction and increased tumor invasiveness (Zhou et al., 2005). Conversely, EMMPRIN neutralizing antibody reduced VEGF and MMP-9 expression (Tang et al., 2006) leading to reduced invasiveness. EMMPRIN may be found as a transmembranal protein or in its secreted form, and both forms can mediate its homophilic interactions (Belton et al., 2008). In this study we show that EMMPRIN is up-regulated by tumor cell-macrophage interactions and its proteolytic cleavage is enhanced in co-culture, resulting in increased amounts of the soluble form. Moreover, we show that the secreted form is sufficient to induce both VEGF and MMP-9, and is pro-angiogenic by itself.

## Methods and materials

### Cells

The human renal carcinoma A498 (ATCC HTB-44), breast carcinoma MCF-7 (ATCC HTB-22) and U937 monocyte-like cells (ATCC CRL-1593) were cultured in RPMI-1640 medium with 10% fetal calf serum (FCS), 1% L-glutamine and antibiotics. The three cell lines were regularly tested for morphological changes and presence of mycoplasma. In some experiments cells were subjected to stimulation with TNFα (1 ng/ml, R&D systems, Minneapolis, MN), incubated with anti-EMMPRIN (LEAF™ Purified anti-human CD147 Antibody, BioLegend, San Diego, CA) or with recombinant EMMPRIN (R&D systems). To avoid possible masking of signals by exogenous stimuli, 10^6^ cells were plated in 24-well plates in RPMI-1640 without FCS and TNFα was added for 48 h. In all co-cultures, tumor cells and U937 cells were plated at a 2:1 ratio. Cell viability was determined using the XTT kit (Biological industries, Beit-Haemek, Israel). The human endothelial cell line EaHy926 (gift of Dr. C. J. Edgell, University of North Carolina, Chapel Hill, NC) was cultured in DMEM with 2% glutamine, 10% FCS, 2% hypoxanthine-aminopterin-thymidine (HAT), and 1% antibiotics.

### ELISA

The human EMMPRIN, MMP-9 and VEGF ELISA kits were performed according to the manufacturer's instructions (R&D systems). Samples were diluted 1:200 for determination of EMMPRIN and MMP-9, and 1:100 for determination of VEGF, according to previous calibration experiments.

### Flow cytometry

10^6^ Cells were centrifuged and re-suspended in RPMI-1640 with 1% FCS, and then incubated with 1 μg of Alexa 647-conjugated anti-human CD147 or with its isotype control (BioLegend) for 30 min at 4°C. After washing, the cells were fixed in 0.1% formaldehyde and analyzed by flow cytometer, (LSRII, BD Biosciences, San Jose, CA). To distinguish between EMMPRIN expression on U937 cells and on tumor cells, A498 and MCF-7 cells were first labeled with 1 μM of Cell Tracker™ Green CMFDA (Life Technologies-InVitrogen, Darmstadt, Germany) according to the manufacturer's instructions, and only then were incubated in the experimental conditions. Dead cells were excluded from the analysis by their forward and sideway light-scattering properties.

### Quantitative real-time PCR

Total RNA was extracted from 10^6^ U937, A498, or MCF-7 cells using the RNA extraction kit (Norgen biotek, Ontario, Canada), and 500 ng were transcribed to cDNA using the High Capacity cDNA Reverse Transcription kit (Applied Biosystems, Foster City, CA). EMMPRIN mRNA expression and its reference gene GusB, or miR-146a and its reference gene U6 were quantified by real-time PCR using the TaqMan assay on demand kit with the StepOne system (Applied Biosystems). The comparative ΔΔC_T_ method was used for relative quantification, and non-stimulated cells served as a calibrator in each experiment, to allow comparison of relative quantity (RQ) between the samples.

### Reverse transfection and inhibition of MIR-146a or EMMPRIN expression

The siPORT *NeoFX* transfection agent (Applied Biosystems/Ambion, Austin, TX) was diluted 1:25 with OPTI-MEM1 medium (Gibco, Invitrogen), combined with 30 nM of the anti-miR-146a inhibitor™ or its Cy3-labeled negative control (anti-miR-NC), or with 5 nM of EMMPRIN siRNA or its negative control (all reagents from Ambion). Solutions were incubated 10 min to allow transfection complexes to form and then dispensed into 24-well plates. 6 × 10^4^ A498 or MCF-7 cells/well were overlaid in suspension over the transfection complexes and gently tilted to evenly distribute the complexes. Cells were incubated at 37°C overnight, followed by replacement with fresh medium and stimulation with TNFα for 48 h. These conditions were calibrated according to the manufacturer's instructions, reaching transfection efficiency of >92%.

### Isolation of EXOSOMES

10^6^ A498 or MCF-7 cells were incubated in single- or co-cultures with 0.5 × 10^6^ U937 cells in the presence of TNFα (1 ng/ml), supernatants were collected and centrifuged at 800 g for 10 min and then at 12,000 g for 30 min to sediment suspended cells. The resulting supernatants were ultra-centrifuged at 110,000 g (Micro-Ultracentrifuge RCM150, rotor S120AT2-0200; Thermo Scientific, Sorvall, Suwanee, GA, USA) for 1.5 h at 4°C to pellet the exosomes. Both pellets and supernatants were evaluated for the presence of EMMPRIN protein by ELISA.

### *In vitro* “wound assay”

EaHy926 monolayers (1 × 10^6^ cells) in 24-well dishes were wounded with a wooden toothpick after overnight incubation, and the line of injury was marked. Detached cells were washed away with medium, and cells were incubated with or without human recombinant EMMPRIN (200 ng/ml) or with 100 μl of supernatants (diluted 1:4 with medium) derived from the siRNA experiments. Images of the field of injury were acquired at the beginning of the experiment and after 48 h. In each experiment, average distances between the two sides of the wound were measured in different locations along the wound (at least 10 locations per field), in day 0 and in day 2, and analyzed with ImagePro plus 4.5 software. The percent change was then calculated relative to day 0.

### *In vivo* plug assay

Liquid Matrigel (0.4 ml) was mixed with 200 ng/ml of human recombinant EMMPRIN and injected subcutaneously into the flank of BALB/c mice. As a control, Matrigel was mixed with serum-free DMEM and injected as above. Matrigel plugs were surgically removed after 7 days and photographed to give visual assessment of angiogenesis. All animal studies were approved by the Animal Care Committee of the Technion.

### Statistical analyses

All values are presented as means ± SE. Significance between two groups was determined using two-tailed unpaired *t*-test. Differences between three or more experimental groups were analyzed using analysis of variance (ANOVA) and the Bonferroni's multiple comparison tests. *P*-values exceeding 0.05 were not considered significant.

## Results

### Co-culture increases the expression of EMMPRIN, MMP-9 and VEGF

Preliminary studies were performed to calibrate the *in vitro* system. TNFα was added to each of the single cell cultures at a concentration of 1 ng/ml, which is similar to the concentration found in the tumor microenvironment (Elamin et al., 2008; Charles et al., 2009; Ali et al., 2012). At this concentration TNFα was sufficient to induce MMP-9, but did not induce cell death, as was estimated by the XTT assay (1.03 ± 0.04, 0.96 ± 0.02, and 0.99 ± 0.05 folds for A498, MCF-7, and U937 cells, respectively, relative to each of the non-stimulated cells). Furthermore, incubation time of 48 h was optimal to observe accumulation of VEGF and MMP-9 in the supernatants. As macrophages may make up as much as 50% of the tumor mass, tumor cells and monocytes were incubated at a ratio of 2:1, as was demonstrated before (Blot et al., 2003; Perske et al., 2010).

**Figure 1:**
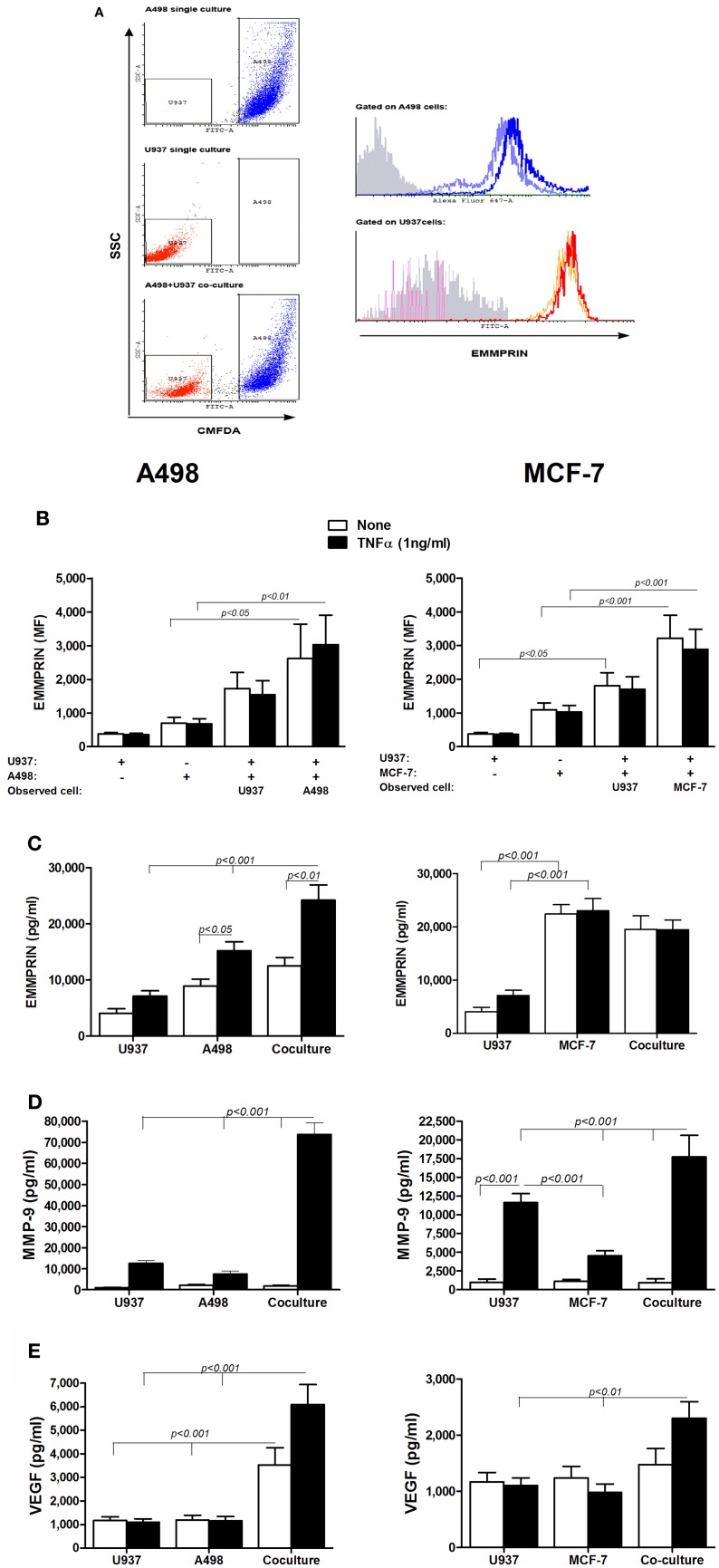
**The effect of co-culture on the secretion of EMMPRIN, MMP-9 and VEGF**. 10^6^ A498 or MCF-7 cells were incubated in a serum-free medium either separately or with 0.5 × 10^6^ U937 cells in co-culture for 48 h, with or without the addition of TNFα (1 ng/ml), and 0.5 × 10^6^ U937 cells were incubated in a serum-free medium with or without the addition of TNFα (1 ng/ml). **(A)** Representative dot plot for the A498 and U937 co-cultures. Light blue and orange histograms—EMMPRIN expression in single cultures of A498 cells and U937 cells, respectively; Blue and red histograms—EMMPRIN expression measured separately on A498 or U937 cells, respectively, incubated in their co-cultures. Gray histogram—isotype control. **(B)** Mean fluorescence (MF) of the membranal expression of EMMPRIN that was evaluated by flow cytometry (*n* = 8). Concentrations of secreted proteins were determined in the supernatants by ELISA for **(C)** EMMPRIN (*n* = 6), **(D)** MMP-9 (*n* = 8) and **(E)** VEGF (*n* = 8).

In all three cell lines examined separately or in co-culture, all of the cells (99.6 ± 0.3%) expressed surface EMMPRIN, however, with different intensities. The U937 cells expressed low levels of membranal EMMPRIN (Figures 1A,B), which increased by 4.5-folds during co-culture with both A498 and MCF-7 cells. Likewise, expression of membranal EMMPRIN on the tumor cells that was 2–3-fold higher in the single cultures than in the U937 cells, was increased during co-culture (by 4-folds and 3-folds in the A498 and MCF-7 cells, respectively, *p* < 0.05 and *p* < 0.001 relative to the singe cultures). TNFα had no effect on the membranal expression of EMMPRIN in all cell types, during separate incubation or in co-cultures. In contrast, TNFα elevated the amounts of secreted EMMPRIN from A498 cells cultured alone (by 2-folds, *p* < 0.05), but not from MCF-7 cells, which already secreted high levels of EMMPRIN (Figure 1B). Co-culture increased the accumulation of secreted EMMPRIN in the A498 experiments (by 3-folds and 1.5-folds relative to the U937 and A498 single cultures, respectively, *p* < 0.001, Figure 1C), although this increase was only additive. In contrast, using the MCF-7 cells, the amounts of the secreted EMMPRIN were not significantly different in the co-cultured cells relative to the single MCF-7 culture, suggesting that this cell line already secreted maximal amounts of EMMPRIN without further stimulation.

In both tumor cell lines and in the monocytic cell line, presence of TNFα was necessary to induce MMP-9 secretion in each of the single cultures, however, the co-cultures significantly elevated this level, synergistically for the A498 cells (5-folds, *p* < 0.001) and additively for the MCF-7 cells (1.6-folds, *p* < 0.001, Figure 1D). Similarly, co-cultures increased the accumulation of secreted VEGF (Figure 1E). In the A498 cells, co-cultures synergistically increased VEGF secretion (by 3-folds, *p* < 0.001 relative to each of the single cultures), and addition of TNFα, which had no effect in the single cultures, further stimulated secretion by 2-folds. In the MCF-7 cells, co-cultures increased the accumulation of VEGF additively only in the presence of TNFα (by 2-folds, *p* < 0.01 relative to each of the single cultures).

### EMMPRIN expression is post-transcriptionally regulated by MIR-146a

**Figure 2:**
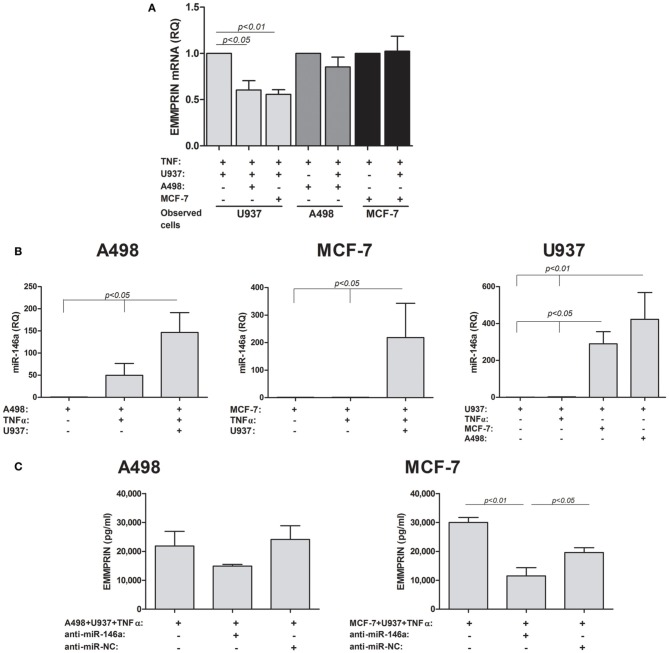
**EMMPRIN is regulated at the post-transcriptional level**. 0.6 × 10^5^ A498 or MCF-7 cells were incubated in single- or co-cultures with 0.3 × 10^5^ U937 cells in the presence of TNFα (1 ng/ml) for 48 h. To allow estimation of mRNA or miRNA levels in each cell type, co-cultured cells were incubated in separate inserts (0.3 μm pore size), thus precluding cell migration between the two compartments. In some of the wells cells were transfected with anti-miR-146a or anti-miR-NC (scrambled negative control). Total RNA was extracted and transcribed to cDNA. **(A)** Expression of the short EMMPRIN mRNA isoform was determined by quantitative real-time PCR amplification (*n* = 4). **(B)** The expression of miR-146a was determined in each of the single and co-cultured cells cell lines (*n* = 3) **(C)** The effect of neutralization of miR-146a in the tumor cell lines on the expression of EMMPRIN protein in co-cultures was determined (*n* = 3).

We next evaluated the expression of EMMPRIN at the RNA levels. EMMPRIN has two known isoforms. The short isoform has only two Ig-like domains, whereas the long isoform has 3 such domains. Preliminary experiments indicated that both isoforms are expressed in the three cell lines, however, the short isoform was the prevalent one (average Ct values of 20 cycles), whereas the long isoform was scarce (average Ct values of 31). We, therefore, evaluated the effect of TNFα and co-culture on the accumulation of EMMPRIN mRNA levels of the short isoform only (Figure 2A). In order to estimate the level of EMMPRIN mRNA in each cell type, co-cultured cells were incubated in separate inserts (membrane of 0.3 μm pore size), so as to preclude cell migration between the two compartments. No significant change was observed in the EMMPRIN mRNA levels in either A498 or MCF-7 cells relative to each of the single cultures, whereas in the U937 cells co-culturing with either A498 or MCF-7 cells reduced these levels by 40 and 45% relative to the single culture (*p* < 0.05 and *p* < 0.01, respectively).

The discrepancy between the elevated protein levels observed before and the unchanged or decreased levels of the mRNA suggested a post-transcriptional regulation of EMMPRIN, which could be mediated through microRNA. We chose to examine the involvement of miR-146a that is a known inflammatory microRNA and has been implicated in tumor-macrophage interactions before (Perske et al., 2010). We could show (Figure 2B) that the expression of miR-146a was elevated by the co-culture relative to each of the non-stimulated single cultures (by 146-folds, ~200-fold, and ~300-folds for A498, MCF-7 and U937, *p* < 0.05). As miR-146a is an inflammatory miRNA, TNFα increased its levels, although not significantly (by 50-folds and 2.5-folds for A498 and U937 cells, respectively), and the co-culture further increased it in the stimulated A498 and U937 cells (by 3-folds and 160-folds, *p* < 0.05), whereas in the MCF-7 cells TNFα did not increase miR-146a expression relative to the non-stimulated cells.

To show that this microRNA regulates the expression of EMMPRIN, we next neutralized its activity by transfecting the tumor cells with its antagomir anti-miR-146a and then co-culturing them with the U937 monocytes in the presence of TNFα (Figure 2C). This neutralization resulted in decreased levels of secreted EMMPRIN protein, which did not reach significance in the A498 cells (by 1.5-folds), but was significantly different (3-folds, *p* < 0.01) for MCF-7 cells.

### Soluble EMMPRIN is generated by a serine protease

**Figure 3:**
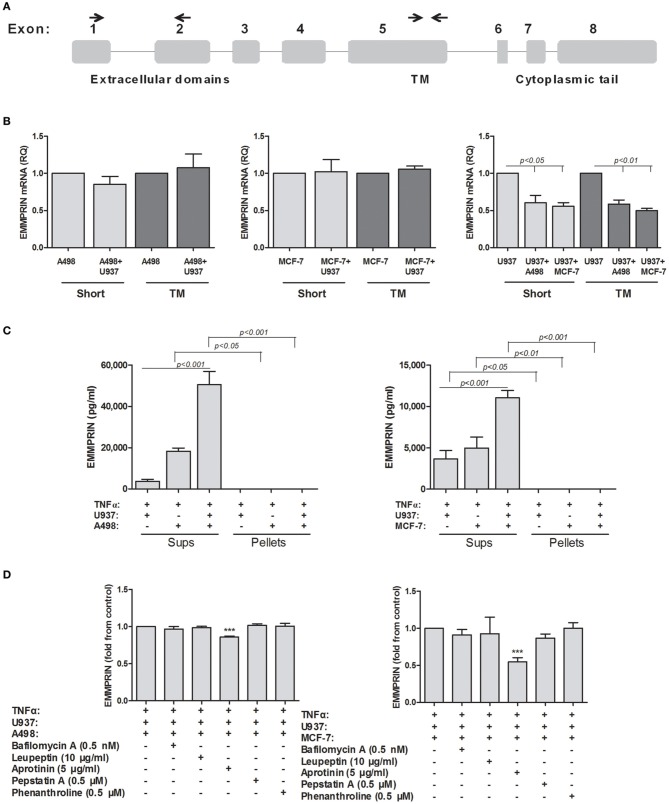
**Possible mechanisms for generating secreted EMMPRIN protein**. 10^6^ A498 or MCF-7 cells were incubated in single- or co-cultures with 0.5 × 10^6^ U937 cells in the presence of TNFα (1 ng/ml) for 48 h. **(A)** A schematic representation of the different exons of the EMMPRIN mRNA. The primers used for the amplification of the transmembranal (TM) or extracellular regions of the short isoform are indicated by small arrows. **(B)** Total RNA was extracted and transcribed into cDNA. Amplification of both the extracellular and transmembranal regions by quantitative real-time PCR was used to assess alternative splicing (*n* = 3). **(C)** Supernatants were collected and ultra-centrifuged as described in the methods. Presence of the EMMRIN protein in exosomes was determined by evaluating its concentrations in both pellets and supernatants by ELISA (*n* = 4). **(D)** Ability of a wide range of protease inhibitors (optimal concentrations indicated) to inhibit EMMPRIN secretion was evaluated by ELISA (*n* = 5).

The way that the secreted form of EMMPRIN is generated remains controversial, where some evidences indicate secretion by exosomes (Keller et al., 2009), and some demonstrate proteolytic cleavage by MMPs (Tang et al., 2004). We investigated several different possibilities for the generation of soluble EMMPRIN. First, we explored whether the co-culture of the tumor cell lines with the U937 monocytes induce alternative splicing of the EMMPRIN mRNA, so that the transmembranal portion of the molecule is deleted and the product cannot be anchored to the membrane. We, therefore, amplified the extracellular and transmembranal regions of the short EMMPRIN mRNA (indicated in Figure 3A) and quantified the transcript by both sets of primers. No change was observed between the extracellular (short isoform) and transmembranal regions of the single- and co-cultures of the A498 and MCF-7 cells (Figure 3B). Likewise, no such change was observed for the U937 cells, despite the reduction in the overall EMMPRIN transcript that was observed before (Figure 2A).

Next, we evaluated the presence of EMMPRIN protein in exosomes, by precipitating the exosomes using ultracentrifugation, an accepted method for exosome isolation (Lahat et al., 2011). However, EMMPRIN was exclusively found in the supernatants and was not sedimented at all after ultracentrifugation, indicating that it is not found in exosomes (Figure 3C).

Finally, we explored the possibility of proteolytic cleavage of the membranal EMMPRIN. To this end, we incubated the tumor cell lines in co-cultures with U937 monocytes in the presence of TNFα, to give rise to maximal secretion of EMMRPIN, and with a wide range of protease inhibitors (Aprotinin, the serine protease inhibitor; Leupeptin, the serine, cysteine and threonine protease inhibitor; Pepstatin A, the aspartyl protease inhibitor; Phenanthroline, the MMPs inhibitor), and with bafilomycin A1, the lysosomal inhibitor which prevents lysosomal acidification. These inhibitors collectively inhibit families of proteases with potential access to membranal EMMPRIN either at the plasma membrane or at the endosomal pathway. Different inhibitors in optimal concentrations, which were determined to be incapable of causing cell death in preliminary experiments (data not shown), were added to the co-cultures and their effect on the secretion of EMMPRIN was evaluated by ELISA. Each value is presented as fold from control (the co-cultured cells in the presence of TNFα) to allow better visualization of the differences exerted by the inhibitors. In both A498 and MCF-7 co-cultures with the monocytic U937 cell, only aprotinin (Figure 3D) caused a significant decrease in secreted EMMPRIN (a 14 and 46% reduction in A498 and MCF-7 co-cultures, respectively, *p* < 0.001).

### EMMPRIN is required for full induction of VEGF and MMP-9

VEGF and MMP-9 are essential for tumor angiogenesis, and can be induced by a myriad of mediators found in tumor microenvironment, including hypoxia, TNFα or EMMPRIN. We used three approaches to demonstrate that EMMPRIN is required for maximal induction of both VEGF and MMP-9.

**Figure 4:**
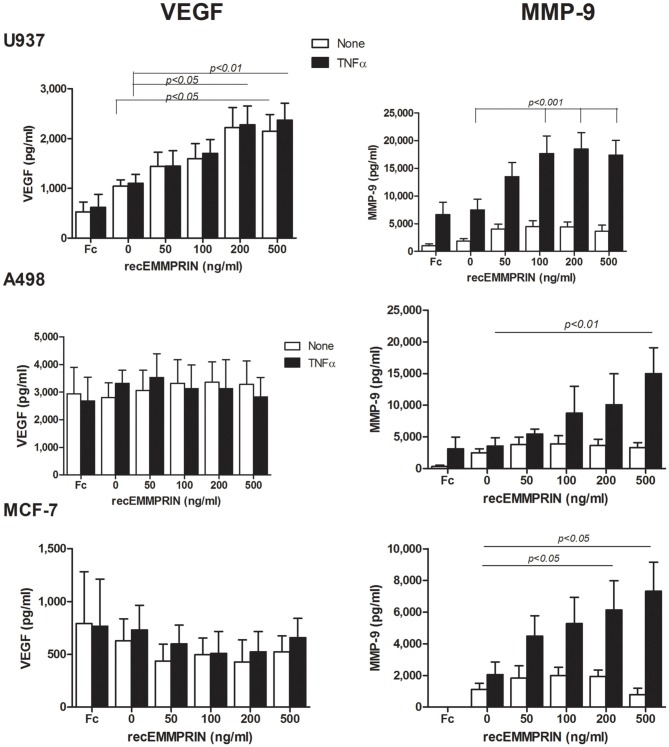
**Effect of recombinant EMMPRIN on the secretion of MMP-9, VEGF, and EMMPRIN**. 5 × 10^5^ A498, MCF-7 or U937 cells were incubated separately in a serum-free medium with or without TNFα (1 ng/ml) for 48 h, and with the addition of increasing amounts of recombinant EMMPRIN or the IgG Fc fragment (at 200 ng/ml). Concentrations of MMP-9 and VEGF were determined in the supernatants using ELISA (*n* = 7).

First, we incubated each of the cell lines, with or without TNFα, with increasing amounts of recombinant EMMPRIN. EMMPRIN alone had no significant effect on the secretion of MMP-9 in all three cell lines (Figure 4), but in the presence of TNFα the high amounts of the recombinant protein increased the secretion of MMP-9 by 2.5-folds from the U937 cells (*p* < 0.001), by 4-folds from the A498 cells (*p* < 0.01) and by 3-folds from the MCF-7 cells (*p* < 0.05), demonstrating a dose response. The secretion of VEGF was not affected by the presence of TNFα, and was significantly increased by the high amounts of the recombinant protein only in the case of the U937 cells (by 2-folds, *p* < 0.01). Since the recombinant EMMPRIN we used is a chimeric product where the IgG Fc fragment is fused to the carboxy-terminus of EMMPRIN, we made sure that this fragment alone does not induce MMP-9 or VEGF secretion from the cells (Figure 4) and used it as a control in all the experiments.

**Figure 5:**
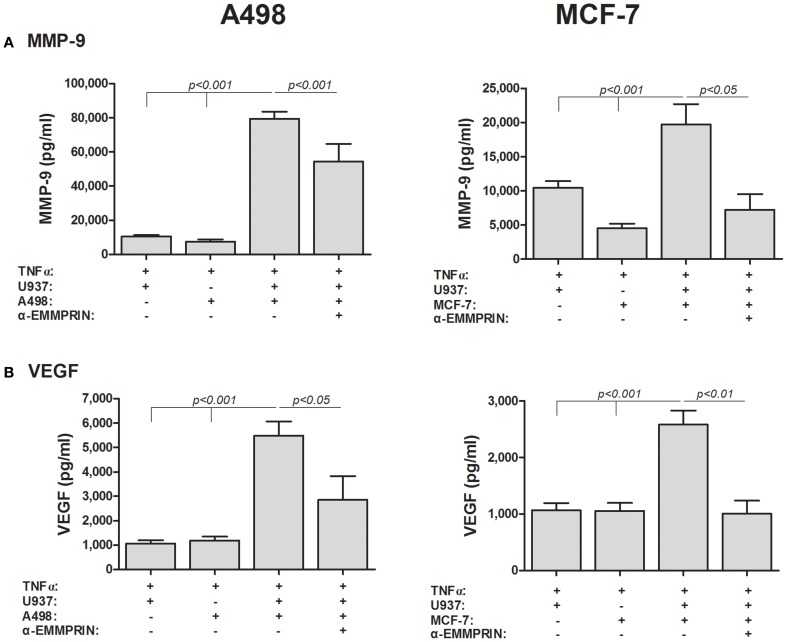
**EMMPRIN neutralization inhibits secretion of VEGF and MMP-9**. 5 × 10^5^ A498 or MCF-7 cells were incubated separately or in co-cultures with 2.5 × 10^5^ U937 cells in a serum-free medium supplemented with TNFα (1 ng/ml) for 48 h, with or without the addition of anti-EMMPRIN (2 ng/ml). Concentrations of **(A)** MMP-9 and **(B)** VEGF were determined in the supernatants using ELISA (*n* = 7).

Secondly, we incubated the TNFα-stimulated cells in co-culture, and neutralized the effects of EMMPRIN by adding its specific antibody (at 2 ng/ml), a concentration that was determined after preliminary calibration experiments. In A498 cells co-cultured with U937 cells, anti-EMMPRIN reduced MMP-9 secretion by 32% (*p* < 0.001) and VEGF secretion by 48% (*p* < 0.05) (Figure 5). Likewise, in MCF-7 cells co-cultured with U937 cells the antibody reduced MMP-9 secretion by 63% (*p* < 0.05) and VEGF secretion by 61% (*p* < 0.01). In the case of MCF-7, the antibody reduced the expression of both MMP-9 and VEGF to levels comparable to those induced by TNFα in the single cultures, whereas in the A498 cells the antibody reduced MMP-9 and VEGF levels only partially, and MMP-9 and VEGF levels were still higher than those found in the TNFα-stimulated single cultures (Figure 5).

**Figure 6:**
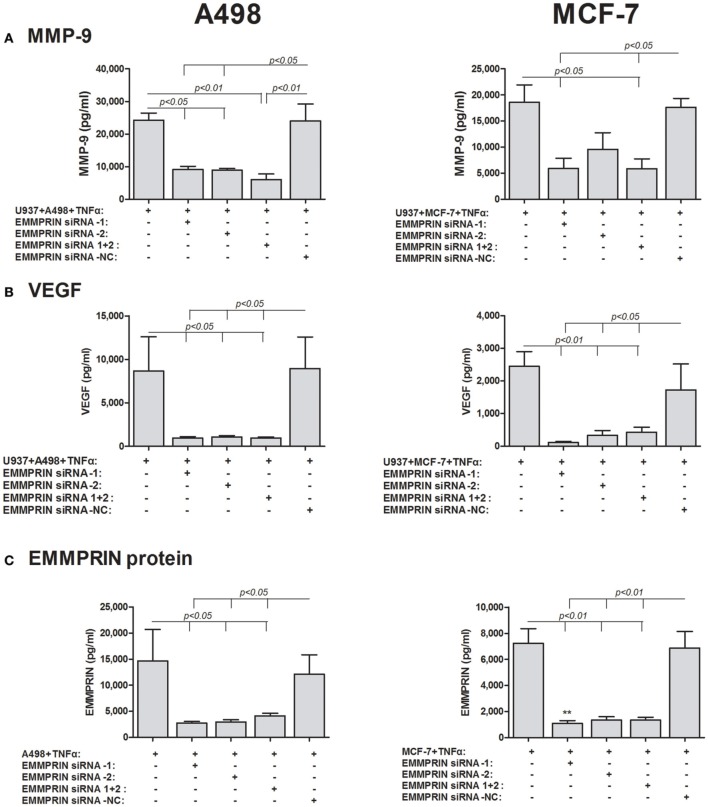
**Effect of EMMPRIN siRNA on the secretion of MMP-9, VEGF and EMMPRIN**. 0.6 × 10^5^ A498 or MCF-7 cells were incubated separately or in co-cultures with 0.3 × 10^6^ U937 cells in a serum-free medium supplemented with TNFα (1 ng/ml) for 48 h. Tumor cells were first transfected with 5 nM of each of the two different EMMPRIN siRNA molecules, their combination or the negative control (NC), or left untreated. Concentrations of **(A)** MMP-9, **(B)** VEGF, and **(C)** EMMPRIN were determined in the supernatants using ELISA (*n* = 5).

Thirdly, we specifically reduced the expression of EMMPRIN in the tumoral cells using EMMPRIN siRNA. To make sure that EMMPRIN was maximally knocked down, we used two different siRNA sequences as well as their combination, and all three possibilities resulted in similar inhibition of EMMPRIN protein production (about 5-folds for both A498 and MCF-7 cells, *p* < 0.05 and *p* < 0.01, respectively, Figure 6C). In A498 or MCF-7 co-cultures with the monocytic U937 cell line, the absence of EMMPRIN resulted in inhibition of VEGF secretion by 8–9-folds and 6–7-folds, respectively, (*p* < 0.05 and *p* < 0.01, respectively, relative to the TNF-stimulated co-cultures without knocking down EMMPRIN, Figure 6B). Likewise, in both A498 and MCF-7 co-cultures, reduced EMMPRIN expression inhibited secretion of MMP-9 by about 3-folds (*p* < 0.05, Figure 6A).

### Soluble EMMPRIN is sufficient to induce MMP-9 and VEGF

**Figure 7:**
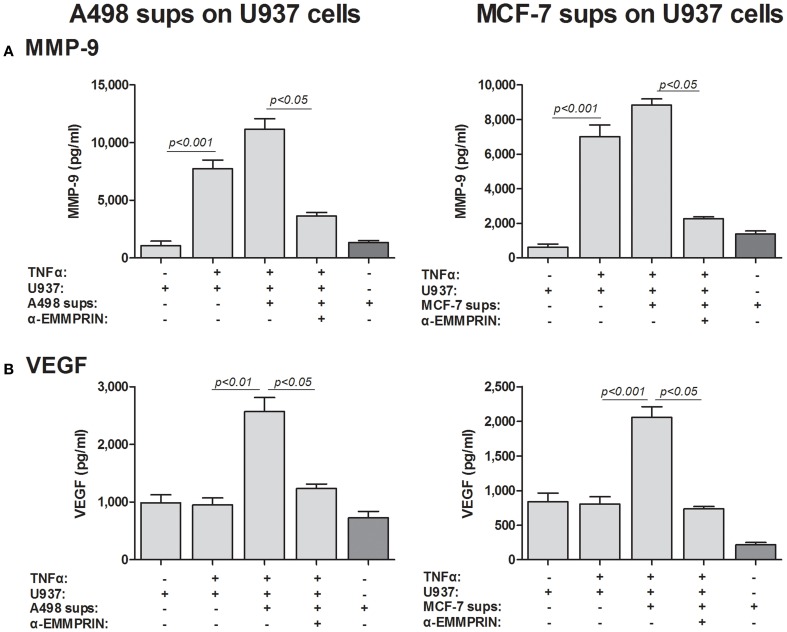
**Effect of cancer cell supernatants on monocyte expression of VEGF and MMP-9 is mediated through soluble EMMPRIN**. 2 × 10^5^ U937 cells were separately incubated in the presence of TNFα (1 ng/ml) for 48 h with or without the addition of diluted (1:4) supernatants derived from A498 or MCF-7 cells, and with the neutralizing anti-EMMPRIN antibody (2 ng/ml). Concentrations of **(A)** MMP-9 and **(B)** VEGF were determined by ELISA (*n* = 5).

**Figure 8:**
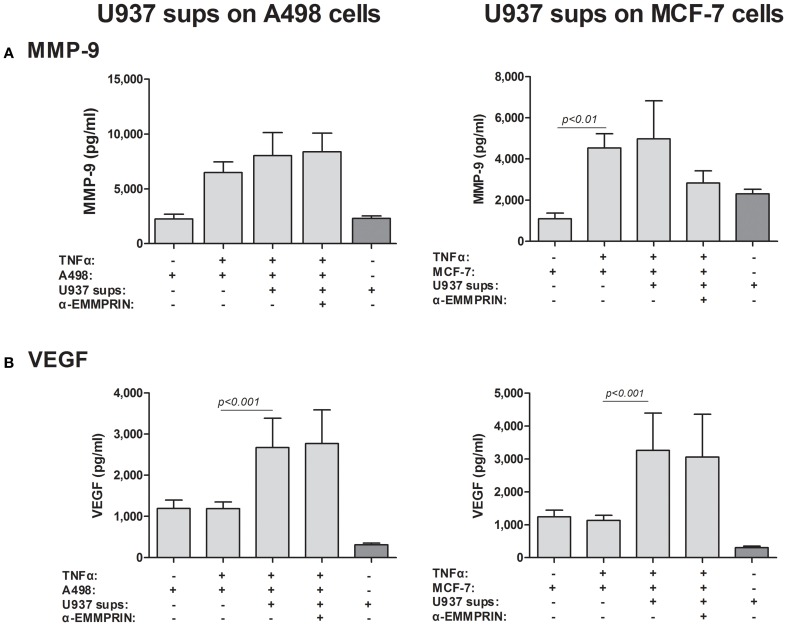
**Effect of monocyte supernatants on cancer cell expression of VEGF and MMP-9**. 2 × 10^5^ A498 or MCF-7 cells were separately incubated in the presence of TNFα (1 ng/ml) for 48 h with or without the addition of diluted (1:4) supernatants derived from U937 cells, and with the neutralizing anti-EMMPRIN antibody (2 ng/ml). Concentrations of **(A)** MMP-9 and **(B)** VEGF were determined by ELISA (*n* = 5).

Although we have shown that EMMPRIN is required for the maximal induction of VEGF and MMP-9 in co-cultures of tumor cells with monocytes, it remains unclear whether the effect is mediated by the membranal or the soluble protein. The dose-dependent effect of the recombinant EMMRPIN (Figure 4) was a first indication that the soluble protein mediates the effect. We further reasoned that soluble EMMPRIN found in the supernatant would be sufficient to induce secretion of VEGF and MMP-9 in the opposite cell type. We therefore, collected sups from TNFα-stimulated single cultures of each of the tumor cells, and incubated them diluted in medium at a ratio of 1:4 with U937 cells, with or without the presence of TNFα (Figure 7). Relative to the TNFα-stimulated U937 single culture, the A498- or MCF-7-derived sups non-significantly increased MMP-9 secretion from U937 cells by 1.44- and 1.26-folds, respectively, but anti-EMMPRIN specifically inhibited it by 3–4-folds (*p* < 0.05). Similarly, the A498- or MCF-7-derived sups increased VEGF secretion from U937 cells by 2.5-folds (*p* < 0.01), and anti-EMMPRIN specifically inhibited it by 2–3-folds (*p* < 0.05). In contrast, the reciprocal incubation of U937-derived sups with both TNFα-stimulated tumor cells (Figure 8) did not affect MMP-9 secretion, and elevated VEGF secretion by about 2.5-folds (*p* < 0.001). However, this effect on VEGF was not specific to EMMPRIN, as indicated by the lack of response to anti-EMMPRIN.

**Figure 9:**
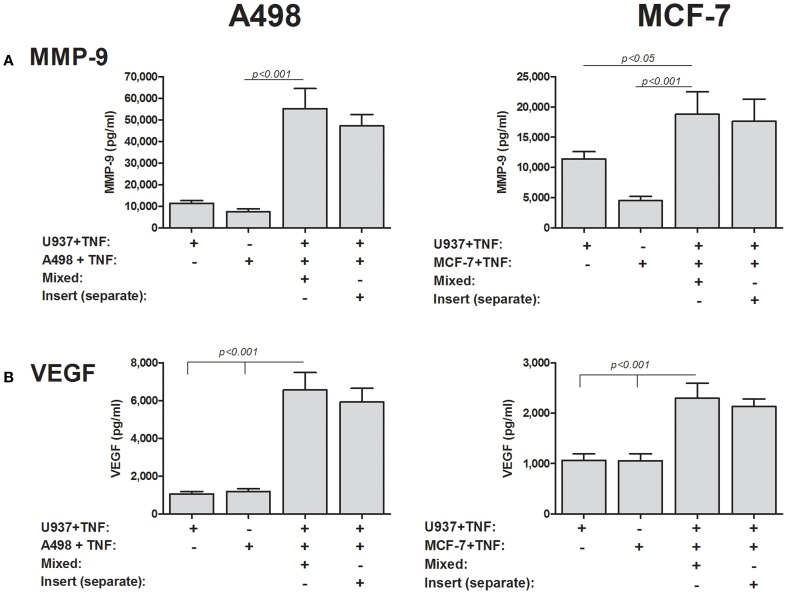
**Cell–cell contact is not required for induction of MMP-9 and VEGF**. 2 × 10^5^ A498 or MCF-7 cells or 1 × 10^5^ U937 cells were incubated with TNFα (1 ng/ml) for 48 h, separately, in a mixed co-culture where cell–cell contact was allowed, or in inserts with a small pore size (0.3 μm) that precluded cell migration and cell–cell contact. Concentrations of **(A)** MMP-9 and **(B)** VEGF were determined by ELISA (*n* = 8).

To finally prove that soluble EMMPRIN can mediate MMP-9 and VEGF induction in co-cultures, we incubated the two cell types in Boyden-modified chambers, where the inserts had a 0.3 μm pore size that prevented cell–cell contact. As before, the co-incubation of the A498 or the MCF-7 tumor cells with the U937 monocytic cell line in a mixture that allows cell–cell contact resulted in increase of both MMP-9 and VEGF in the supernatants, whereas the separate incubation of the cells in the inserts did not change this effect (Figure 9).

### Soluble EMMPRIN is pro-angiogenic

**Figure 10:**
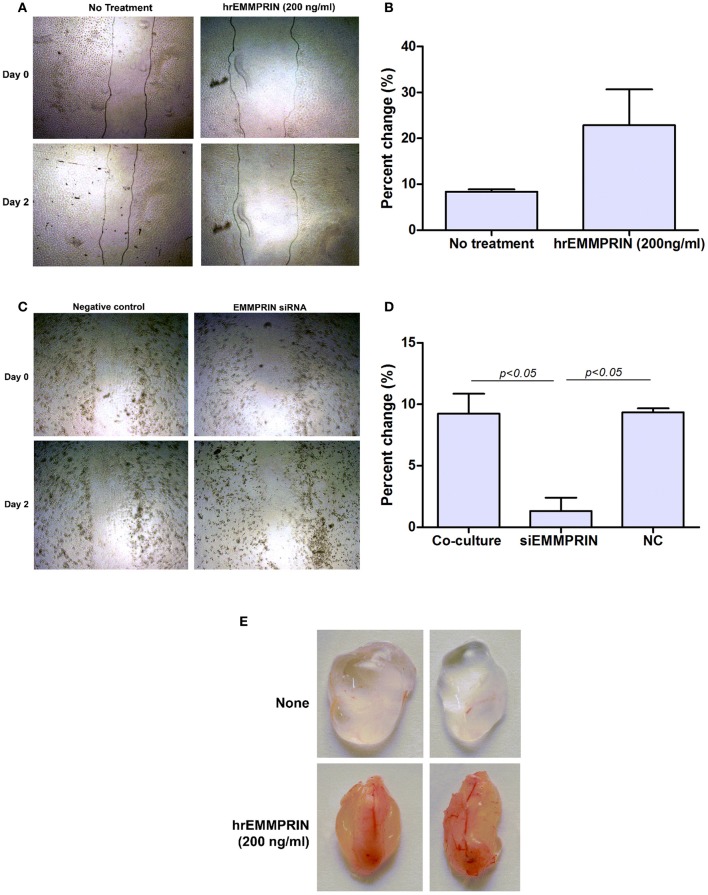
**Soluble EMMPRIN has pro-angiogenic properties**. 2 × 10^6^ Eahy926 cells were grown to confluency in 24-well plates, and a wound assay was performed by making a scratch, as described in the methods. **(A)** A representative photograph of the scratched area in a wound assay at the beginning (0 h) and at the end (48 h) of the experiment (magnification X40) while adding recombinant EMMPRIN (200 ng/ml). Gray lines depict the cell front. **(B)** Rate of wound closure was measured by comparing the distance between the two sides of the scratch using image analysis software at the beginning and at the end of the experiment, and calculating the percent of change (*n* = 3). **(C)** A representative photograph of scratched area in a wound assay when adding diluted (1:4) supernatants derived from the co-cultures or co-cultures where A498 cells were first transfected with EMMPRIN siRNA or its negative control (NC), and **(D)** analysis of its rate of wound closure (*n* = 3). **(E)** Matrigel plug assay was carried out as described in the methods, and plugs were removed and photographed after 7 days.

In addition to the ability of EMMPRIN to induce VEGF and MMP-9, we asked if EMMPRIN has direct pro-angiogenic effect on endothelial cells. Since endothelial cells migrate as one sheet, and migration and proliferation are inseparable, the distances between the two sides of the wound and their enhanced rate of closure over time reflect a pro-angiogenic activity. Figures 10A,B demonstrate that recombinant EMMPRIN (200 ng/ml) increased migration/proliferation of EaHy926 cells by 2.7-folds, although this trend did not reach significance. In a complementary experiment (Figures 10C,D), supernatants derived from A498 tumor cells that were transfected with EMMPRIN siRNA demonstrated a 7-fold (*p* < 0.05) decreased ability to repair the wound compared to untreated supernatants. Similar results were observed for MCF-7-derived supernatants (data not shown), suggesting a requirement for EMMPRIN presence.

To evaluate the pro-angiogenic effects of EMMPRIN *in vivo* we used the Matrigel plug assay, where liquefied Matrigel mixed with medium or with recombinant EMMPRIN (200 ng/ml) was injected subcutaneously. The plugs were harvested after 7 days and their gross morphology is presented (Figure 10E). The control plugs are clear and transparent with almost no visible blood vessels, whereas plugs that were mixed with recombinant EMMPRIN are opaque, reddish and clearly show formation of functional blood vessel network, strongly suggesting increased angiogenesis.

## Discussion

EMMPRIN has been shown before to be pro-angiogenic, through its ability to induce MMPs and VEGF. Here we demonstrate some novel points: (a) Expression of EMMPRIN is increased in tumor cells upon co-culturing with macrophages; (b) EMMPRIN is post-transcriptionally regulated by miRNA-146a; (c) Soluble EMMPRIN retains its biological activity, is required for maximal induction of both VEGF and MMP-9, and is generated by a serine protease that is yet to be identified; (d) soluble EMMPRIN alone has a pro-angiogenic activity and a direct effect on endothelial cells both *in vitro* and *in vivo*.

Enhanced EMMPRIN expression has been described before in many types of tumor tissues, but was usually not accompanied by detection of EMMPRIN expression on stromal cells (Zhong et al., 2008; Liang et al., 2009; Omi et al., 2012; Pinheiro et al., 2012; Lu et al., 2013), suggesting that stromal cell expression of EMMPRIN *in vivo* was very low and below the levels of antibody detection. Studies *in vitro* demonstrated that tumor cells enhance their expression of EMMPRIN when co-cultured with other cell types, such as fibroblasts (Tang et al., 2004; Sato et al., 2009) or endothelial cells (Caudroy et al., 2002; Bougaten et al., 2009), but expression of EMMPRIN in monocytes/macrophages co-cultured with tumor cells was hardly investigated. Here we show that EMMPRIN is constitutively expressed, although in low levels, on both cell types. The interactions of monocytes with tumor cells in co-cultures *in vitro* lead to increased membranal expression of the EMMPRIN protein in both cell types, but mostly in the tumor cells. Thus, in our system monocytes behave similarly to other stromal cells, and it is the tumor cells that are mostly responsible for the overexpression of EMMPRIN.

The molecular mechanism responsible for this elevated expression of EMMPRIN in co-cultures remains unknown, although EGFR and angiotensin II were implicated in its expression in fibroblasts and macrophages, respectively (Weidle et al., 2010; Yang et al., 2010), but not in tumor cells. We have shown that EMMPRIN mRNA in the tumoral cell lines remains unchanged, and has even decreased in the U937 cells, whereas the protein expression was enhanced. This suggested post-transcriptional or translational regulation, and placed microRNA as possible regulators. We have chosen to look at miR-146a, as it plays a major role in both inflammatory processes and in regulation of cancer (Baltimore et al., 2008), and was previously implicated in the regulation of tumor cell-macrophage interactions (Perske et al., 2010). We first demonstrated that miR-146a is increased both in the two tumor cells and in the monocytic cells during co-culture, and then that its neutralization specifically inhibited secretion of EMMPRIN protein. These results further support a role for miR-146a in the interactions between tumor cells and macrophages, and suggest that increased miR-146a enhances, rather than inhibits translation of EMMPRIN. This was unexpected, as microRNAs usually inhibit protein translation. However, it is possible that miR-146a could indirectly regulate another protein that either controls EMMPRIN translation or interacts with EMMPRIN to enhance its degradation. Both possibilities may result in enhanced EMMPRIN expression. Alternatively, since different algorithms (e.g., miRanda, TargetScan) predict that miR-146a can directly bind to EMMPRIN mRNA, it is possible that miR-146a cooperates with other microRNAs, resulting in complex and non-linear effects. It is also noteworthy that microRNAs do not always inhibit translation, and in viruses they have been shown to regulate their enhanced replication, as is the case for the liver-specific miR-122 and hepatitis C virus (Jopling, 2008), suggesting an additional mode of action for microRNAs. The question of which signal elevates the expression of miR-146a in co-culture in both cell types merits further investigation.

In addition to the elevated membranal expression of EMMPRIN, co-cultures also increase soluble EMMPRIN, particularly in the A498 and U937 co-cultures. Previous studies have suggested three possible mechanisms that may generate soluble EMMPRIN. One mechanism is the generation of exosomes from multivesicular bodies undergoing exocytosis that include EMMPRIN (Keller et al., 2009). Another possibility is the secretion of EMMPRIN in microvesicles that are shed off from the surface, particularly from lipid rafts (Millimaggi et al., 2007). This microvesicle-associated EMMPRIN could enhance MMPs production in endothelial cells, as well as increase their migration and ability to form tube-like structures (Millimaggi et al., 2007). A third mechanism for the secretion of EMMPRIN suggests that the surface protein is cleaved by a protease. In fact, MT1-MMP was found to cleave EMMPRIN in the linker region connecting the two Ig-like domains, leading to a reduced ability of the surface protein, but not the secreted protein, to induce MMPs in fibroblasts (Egawa et al., 2006). Our results rule out the possibility of alternative splicing yielding a soluble EMMPRIN protein, as well as the formation of relatively large structures such as microvesicles or exosomes that carry EMMPRIN, as EMMPRIN was not found in the pellets after ultracentrifugation. Our use of a wide range of protease inhibitors ruled out the involvement of most proteases. Specifically, the use of phenanthroline which inhibits zinc metalloproteinases such as MT1-MMP, suggested that MMPS may not be involved in the proteolytic cleavage of EMMPRIN. In contrast, we found that aprotinin, which inhibits several serine proteases, could decrease the accumulation of EMMPRIN in the supernatants, indicating that a serine protease that is yet to be identified is responsible for a shedding off of surface EMMPRIN in our system. The identification of the specific serine protease awaits further investigation, and plasmin or kalikrein, which are inhibited by aprotinin, seem like good candidates.

We also demonstrate that soluble EMMPRIN, whether synthetic (recombinant EMMPRIN) or produced in our co-culture system, was able to induce both MMP-9 and VEGF from the U937 monocytic cells. The experiments where recombinant EMMPRIN, supernatants derived from one cell type and added to another cell type and cells co-cultured using 0.3 μm inserts—all proved that soluble EMMPRIN was sufficient and that it retained its pro-angiogenic activity. Directionality of EMMPRIN activity was disclosed in the experiments where A498- or MCF-7-derived supernatants were added to U937 cells and demonstrated increased MMP-9 and VEGF secretion that was reduced by anti-EMMPRIN. The reciprocal experiment where U937-derived supernatants were added to the tumor cells resulted in increased VEGF, but not MMP-9 secretion, and even this was not specific, as anti-EMMPRIN could not inhibit this effect. It is also noteworthy that other factors found locally in the tumor microenvironment could induce MMP-9 and VEGF. Among these factors, hypoxia and presence of pro-inflammatory cytokines (e.g., TNFα) can induce VEGF and MMP-9 through the activity of the HIF and NF-κB transcription factors (Pages and Pouyssegur, 2005; Yan and Boyd, 2007). EMMPRIN is not redundant, however, and it has a role in maximizing secretion of VEGF and MMP-9, as we show by specifically targeting EMMPRIN, either by siRNA or by anti-EMMPRIN.

Thus, increased tumoral EMMPRIN expression results in generation of soluble EMMPRIN, which probably binds to its ligand on monocytes (either a yet unknown ligand, or EMMPRIN itself through homophilic interactions), and induces secretion of MMP-9 and VEGF from monocytes. This is also in agreement with the fact that monocytes and macrophages are better producers of these proteins than tumor cells.

Finally, EMMPRIN pro-angiogenic activity is not limited to induction of MMP-9 and VEGF. Recent studies have shown that EMMPRIN may directly contribute to the regulation of the angiogenic process by up-regulating soluble forms of VEGF and VEGFR2 in endothelial cells (Bougaten et al., 2009; Pinheiro et al., 2012). We therefore examined the effects of EMMPRIN on endothelial cells *in vitro* using the wound assay. We show that recombinant EMMPRIN could accelerate the rate of wound closure, and when EMMPRIN expression in tumor cells was silenced by siRNA, wound repair was inhibited. Likewise, addition of EMMPRIN to matrigel plugs resulted in growth of blood vessels into the plug, demonstrating that EMMPRIN directly affects endothelial cells *in vivo* as well. Thus, EMMPRIN is directly involved in endothelial migration/proliferation, and the mechanisms that are responsible for this action should be further explored.

In conclusion, we show that co-cultures of tumor cells and macrophages induce EMMPIN expression, as well as production of MMP-9 and VEGF, mostly by the macrophages. Secreted EMMPRIN, which is generated in our system by a serine protease that is yet to be identified, is functional and sufficient to stimulate macrophages to produce VEGF and MMP-9. Furthermore, EMMPRIN has an additional direct pro-angiogenic effect on endothelial cells, suggesting that in the tumoral microenvironment angiogenesis is orchestrated by the effects of EMMPRIN in this triage of tumor cell-macrophage-endothelial cell interactions, and emphasizing the importance of such interactions. Additional questions, such as the deciphering of the signaling pathway that is responsible for the increased EMMPRIN expression and enhanced miR-146a expression merit further investigation.

### Conflict of interest statement

The authors declare that the research was conducted in the absence of any commercial or financial relationships that could be construed as a potential conflict of interest.
